# Targeting TRP channels: recent advances in structure, ligand binding, and molecular mechanisms

**DOI:** 10.3389/fnmol.2023.1334370

**Published:** 2024-01-11

**Authors:** Jian Huang, Aron Korsunsky, Mahdieh Yazdani, Jianhan Chen

**Affiliations:** ^1^Department of Chemistry, University of Massachusetts, Amherst, MA, United States; ^2^Modeling and Informatics, Merck & Co., Inc., West Point, PA, United States

**Keywords:** Transient Receptor Potential Channels, TRP channels, TRP structures, ligand binding sites, activation and gating mechanism, drug design, drug targets

## Abstract

Transient receptor potential (TRP) channels are a large and diverse family of transmembrane ion channels that are widely expressed, have important physiological roles, and are associated with many human diseases. These proteins are actively pursued as promising drug targets, benefitting greatly from advances in structural and mechanistic studies of TRP channels. At the same time, the complex, polymodal activation and regulation of TRP channels have presented formidable challenges. In this short review, we summarize recent progresses toward understanding the structural basis of TRP channel function, as well as potential ligand binding sites that could be targeted for therapeutics. A particular focus is on the current understanding of the molecular mechanisms of TRP channel activation and regulation, where many fundamental questions remain unanswered. We believe that a deeper understanding of the functional mechanisms of TRP channels will be critical and likely transformative toward developing successful therapeutic strategies targeting these exciting proteins. This endeavor will require concerted efforts from computation, structural biology, medicinal chemistry, electrophysiology, pharmacology, drug safety and clinical studies.

## Introduction

Ion channels are integrated membrane proteins that facilitate and regulate the passage of ions through membranes (Hille, 2001). Their activities are controlled by various cellular stimuli including chemical ligands, voltage, temperature, mechanical force and others (Keynes, 1975; Hebert, 1998; Minor, 2010). Dysfunction or mis-regulation of ion channels can lead to a plethora of diseases (Hübner and Jentsch, 2002; Zaydman et al., 2012), and they are considered one of the most important classes of drug targets (Kaczorowski et al., 2008; Clare, 2010; Bagal et al., 2013; Santos et al., 2017; Hutchings et al., 2019). Transient receptor potential (TRP) channels, in particular, are a large and diverse family of ion channels second in size only to potassium channels (Gees et al., 2012; Cao, 2020). They play critical roles in sensory perception and possess polymodal activation by various physical and chemical stimuli (Nilius and Owsianik, 2011). There are 27 members in the human TRP ion channel superfamily. They can be further divided into six subfamilies based on sequence homology, namely, TRPC1-7 (canonical), TRPV1-6 (vanilloid), TRPM1-8 (melastatin), TRPA1 (ankyrin), TRPML1-3 (mucolipin), and TRPP2-3 (polycystins) (Montell et al., 2002). Note that the sequence-based classification does not necessarily cluster TRP channels with the same or similar functionalities–members within one subfamily can have distinct functions. TRP channels are widely expressed in most cells, tissues and organs with varying expression patterns among members (Nilius and Owsianik, 2011). For example, TRPC, TRPA, TRPM are primarily localized in plasma membrane, whereas TRPML and TRPP channels locate in the cytosolic compartments due to their C-terminal endoplasmic reticulum retention-signaling domain.

As multifunctional signaling proteins, TRP channels can sense a wide range of external and internal stimuli and trigger downstream physiological responses (Clapham, 2003; Voets et al., 2005). While the functions of some TRP channels have been well studied, many others remain insufficiently characterized, especially at the molecular level. For example, TRPV1 has a significant role in thermoregulation (Romanovsky et al., 2009; Szolcsányi, 2015), TRPM8 plays a central role in cold sensing (McKemy et al., 2002; Peier et al., 2002; Brauchi et al., 2004; Bautista et al., 2007), TRPA1 could serve as a sensor for pain, noxious cold temperature, environmental irritants, cellular stress and tissue damage (Caspani and Heppenstall, 2009; Viana, 2016; Meents et al., 2019; Souza Monteiro de Araujo et al., 2020) and TRPV5 and TRPV6, two epithelial calcium channels, are responsible for Ca^2+^ reabsorption and thus play a key role in calcium homeostasis (van Abel et al., 2005; van Goor et al., 2017; Khattar et al., 2022; Walker and Vuister, 2023). Overall, due to their important sensory perception roles, studies of the physiological function, activation and regulation of TRP channels have been and will continue to be a hot spot in biological and biomedical research.

With their widespread expression in the human body and extensive involvement in various key psychological and pathological processes (Nilius and Owsianik, 2011), TRP channels are attractive therapeutic targets for treatment of both acquired and hereditary channelopathies (Moran et al., 2011; Fallah et al., 2022; Koivisto et al., 2022). Many traditional natural products from plants and animals have been discovered to target TRP channels. For example, capsaicin from Capsicum and resiniferatoxin from resin spurge are activators of TRPV1, cannabinoids from Cannabis activates TRPV2, menthol from mint can target TRPM8 and TRPV3, and various pungent ingredients from wasabi, mustard, radish activate TRPA1. These compounds have been well-curated in several seminal review papers (Calixto et al., 2005; Vetter and Lewis, 2011; Zhang et al., 2019). These examples also highlight great potentials in exploiting natural products for targeting TRP channels. Many drug candidates, either from natural or synthetic origins, are currently in clinical trials, targeting various TRPVs as well as TRPA1 and TRPM8 channels (Moran and Szallasi, 2018; Iftinca et al., 2021). Furthermore, high resolution structures are now available for all subfamilies at multiple functional states, providing a solid basis for rational approaches toward targeting these proteins (Cao, 2020; Huffer et al., 2020; Diver et al., 2022). Yet, significant gaps remain in the current understanding of the activation and regulation of TRP channels at the molecular level. In this review, we summarize the therapeutic potential of TRP channels as well as recent advances in structural studies of TRP channels, with an emphasis on known ligand binding sites and mechanistic features of TRP channel gating and regulation. We also discuss the perspective on how understanding the molecular mechanisms can help to advance therapeutics and drug development targeting TRP channels.

## Pathological and therapeutic roles of TRP channels

Hereditary mutations in TRP channels can cause a variety of channelopathies, which is not surprising given their important regulatory roles in membrane excitability of sensory neurons and cellular ion homeostasis (Yue and Xu, 2021). For example, TRPV4, which is involved in the most well-documented mutation-induced inheritable channelopathies, is directly linked to peripheral neuropathies, skeletal dysplasia and arthropathy with varied phenotypes and syndromes (Dai et al., 2010; Nilius and Owsianik, 2010; Nilius and Voets, 2013). Currently reported TRP hereditary channelopathies are summarized in Table 1, highlighting the importance of TRP channels as drug targets. Direct modulation of the activities of TRP channels through drugs has also been pursued as an effective strategy to intervene the progressions of pain, respiratory disease, cancer and diabetes (Santoni and Farfariello, 2011; Brederson et al., 2013; Colsoul et al., 2013; Shapovalov et al., 2016; Belvisi and Birrell, 2017). The current status of drug discovery and clinical trials of TRP channels has been well-covered in recent reviews (Yue and Xu, 2021; Koivisto et al., 2022). Herein, we will focus on the most well-known TRP-related acquired diseases – pain and respiratory diseases.

**TABLE 1:** TRP-related hereditary channelopathies.

Member	Channelopathies	References
TRPA1	Familial episodic pain syndrome (GOF)	Kremeyer et al., 2010
TRPV3	Olmsted syndrome (GOF)	Lin et al., 2012; Duchatelet et al., 2014; Ni et al., 2016
TRPV4	Autosomal dominant skeleto-dysplasia brachyolmia type 3 (GOF) congenital distal spinal muscle atrophy (GOF) Charcot-Marie-Tooth disease type 2C (GOF) familial digital arthropathy brachydactyly (LOF) familial digital arthropathy brachydactyly (LOF) parastremmatic dysplasia spondylo-epimetaphyseal dysplasia maroteaux pseudo-Morquio type 2 spondylometaphyseal dysplasia Kozlowski type scapuloperoneal spinal muscular atrophy (GOF)	Thoroughly reviewed in Dai et al. (2010), Nishimura et al. (2012), and Nilius and Voets (2013)
TRPV5	Kidney stone (LOF)	Khaleel et al., 2015; Oddsson et al., 2015; Wang et al., 2017; Ali et al., 2022
TRPV6	Chronic pancreatitis (LOF)	Masamune et al., 2020; Zou et al., 2020
	Transient neonatal hyperparathyroidism (LOF)	Burren et al., 2018; Suzuki et al., 2018, 2020; Yamashita et al., 2019; Almidani et al., 2020; Mason et al., 2020
	Kidney stone (GOF)	Suzuki et al., 2008
TRPC6	Focal and segmental glomerulosclerosis (GOF)	Winn et al., 2005
	Idiopathic pulmonary artery hypertension	Yu et al., 2009
TRPM1	Congenital stationary night blindness (LOF);	Audo et al., 2009; Li et al., 2009; van Genderen et al., 2009
TRPM2	Western Pacific Amyotrophic Lateral Sclerosis and Parkinsonism Dementia	Hermosura et al., 2008
TRPM3	Developmental and epileptic encephalopathies (GOF)	Dyment et al., 2019; de Sainte Agathe et al., 2020; Van Hoeymissen et al., 2020; Zhao S. et al., 2020
TRPM4	Brugada syndrome	Liu et al., 2013; Gualandi et al., 2017
	Progressive symmetric erythrokeratoderma (GOF)	Wang et al., 2019
	Congenital long QT syndrome (LOF)	Hof et al., 2017
	Inherited cardiac conduction defects, including progressive familial heart block type 1 (GOF), childhood atrioventricular block	Kruse et al., 2009; Liu et al., 2010; Daumy et al., 2016; Syam et al., 2016; Xian et al., 2018; Janin et al., 2019; also reviewed in Abriel et al., 2012
TRPM6	Familial hypomagnesaemia with secondary hypocalcemia (LOF)	Walder et al., 2002
TRPM7	Guamanian amyotrophic lateral sclerosis and parkinsonism dementia (LOF)	Hermosura et al., 2005
TRPML1	Mucolipidosis type IV (LOF)	Bassi et al., 2000; Sun et al., 2000
TRPML3	Varitint-waddler (Va) deafness (GOF) (in mice)	Xu et al., 2007; Cuajungco and Samie, 2008; Nagata et al., 2008
TRPP2	Autosomal dominant polycystic kidney disease (LOF)	Mochizuki et al., 1996; Wu and Somlo, 2000

GOF, gain of function; LOF, loss of function.

Some TRP channels, such as TRPV1-4, TRPA1 and TRPM8, are richly expressed in sensory neurons (Mickle et al., 2015), and are prime analgesic targets to eliminate pain sensation (Dai, 2016; Moran and Szallasi, 2018). It has been known that both agonists and antagonists of TRPV1 could silence TRPV1-mediated nociception due to its prolonged desensitization after applying agonists (Noto et al., 2009; Chung and Campbell, 2016; Bonezzi et al., 2020). Downregulating or antagonizing TRPA1 has been shown to reduce cold hyperalgesia in nerve injury models (Obata et al., 2005; Katsura et al., 2006; Caspani et al., 2009; Staaf et al., 2009), mechanical allodynia (Eid et al., 2008; Kerstein et al., 2009; Kwan et al., 2009; Wei et al., 2011; Zappia et al., 2017), and painful diabetic neuropathy (Koivisto et al., 2012) and chemotherapeutic-induced peripheral neuropathy (Staff et al., 2017). Antagonists of TRPM8 have been documented in the treatment of chronic pain and migraine (Weyer and Lehto, 2017). Some natural agonists of TRPM8, such as menthol, have been used for centuries due to their analgesic effects (Patel et al., 2007). These and other TRP channels involved in pain sensation and relief have been extensively reviewed in many seminal reviews (Willis, 2009; Brederson et al., 2013; Fernández-Peña and Viana, 2013; Dai, 2016; González-Ramírez et al., 2017; Moran and Szallasi, 2018; Souza Monteiro de Araujo et al., 2020), which speaks volume to the importance of these ion channel’s role in nociception and the great promise of TRP-targeting drugs in the treatment of pain of various natures.

Interestingly, many nociceptive TRP channels are also expressed widely in sensory neurons that innervate the airway as well as in non-neuronal cells in the lung including structural and immune cells (Belvisi and Birrell, 2017). These channels thus play important roles in the pathophysiology of respiratory diseases [such as asthma and chronic obstructive pulmonary disease (COPD) and chronic refractory cough] (Grace et al., 2014; Koivisto et al., 2022). Antagonizing TRPV1, TRPA1, and TRPV4 was shown to have anti-coughing effects in animal models (Andrè et al., 2009; Khalid et al., 2014; Mukhopadhyay et al., 2014; Bonvini et al., 2016; Mason et al., 2020, p. 4). Airway hypersensitivity, as a respiratory symptom of asthma, can be suppressed by TRPV1 and TRPA1 inhibitors (Raemdonck et al., 2012; Baker et al., 2016). TRPV4 has been frequently linked to pulmonary diseases including acute lung injury, pulmonary edema formation, and pulmonary hypertension, due to its role of sensing osmolarity to regulate the pulmonary capillary permeability (Goldenberg et al., 2015b; Rosenbaum et al., 2020). Inhibition of TRPV4 has also been suggested to be a promising therapeutic route for treating acute lung injury/acute respiratory distress syndrome (ARDS) (Goldenberg et al., 2015a), and more recently for treating COVID-19 patients with lung edema (Kuebler et al., 2020).

The therapeutic potential of TRP channels for other acquired diseases has also been reported. Pharmacological inhibition of TRPM2 shows beneficial effects toward ischemia/reperfusion (I/R) injury in brain, heart and kidney (Zhan et al., 2016). Inhibition of TRPCs, such as TRPC4 and TRPC5, have anxiolytic and antidepressant effects in mice, which could potentially be used for treatment of anxiety disorders (Just et al., 2018). Many TRPs are also intimately connected to itching (Tóth et al., 2015), cardiovascular diseases (Watanabe et al., 2008; Yue et al., 2015), kidney diseases (Hsu et al., 2007; Chubanov et al., 2017), diabetes (Colsoul et al., 2013; Zsombok and Derbenev, 2016), and cancers (Lehen’kyi and Prevarskaya, 2011; Santoni and Farfariello, 2011; Shapovalov et al., 2016; Yang and Kim, 2020).

Developing drugs for TRP-related acquired channelopathies requires deeper understanding of the signaling pathways or the interaction/regulation networks of the TRP channels. For example, TRPV1 antagonists and agonists seemingly have similar therapeutic effects for pain relief (Moran and Szallasi, 2018; Iftinca et al., 2021). The implication follows that developing both inhibitors and activators for TRP channels allows for dealing with complicated syndromes with different therapeutic strategies and/or to minimize side-effects. Cautions, however, should be taken when interpreting the therapeutic effects of agonists or antagonists on these channels. For example, menthol, a known TRPM8 activator, has been used as an antitussive drug, but its mechanism of action may not derive from TRPM8 activation as menthol can also interact with TRPA1 (Karashima et al., 2007). Complication due to promiscuity of various antagonists and agonists toward TRP channels has to be considered, which will likely benefit from better understanding of the molecular mechanism of interaction and regulation of the ligand.

## Recent progresses in TRP channel structure and ligand binding

The first high-resolution TRP channel structure was not determined until 2013, when the structure of TRPV1 was resolved at near-atomic resolution thanks to breakthroughs in cryo-electron microscopy (cryo-EM) (Cao et al., 2013; Liao et al., 2013). This landmark work ushered in a new era in structural biology, where cryo-EM can now be readily applied to obtain high-resolution structures of membrane proteins and other complex bio-macromolecules (Cao, 2020; Diver et al., 2022). Ten years on, there are over 350 structures of TRP channels deposited in the Protein Data Bank (PDB) as of October 2023 (Supplementary Appendix Table 1). At least one structure exists for all the TRPVs, most TRPMs, TRPC3-6, TRPA1, all TRPMLs and TRPP1-3 members. For many TRP channels, structures are available in multiple functional and/or ligand-bound states (either agonists or antagonists), especially those within the TRPV, TRPM and TRPA subfamilies (Supplementary Appendix Table 1). These structures have provided crucial insights into the molecular basis of ion conductance, activation and regulation of TRP channels.

**FIGURE 1:**
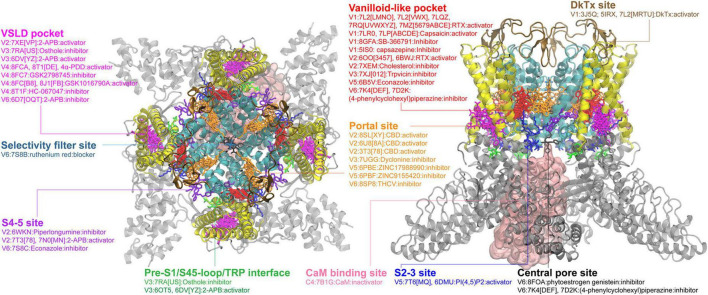
Ligand binding sites in TRPV channels. The TRPV1 structure in complex with DkTx (PDB: 3J5Q) is used for visualizing the binding sites, with the pore region, the VSLD region and cytosolic domain colored in cyan, yellow and gray, respectively. All other structures were first structurally aligned using US-align (Zhang et al., 2022) to allow the clustering of all ligands observed. The major binding pockets or interfaces in the TRPV channels are highlighted using clusters of ligands shown in different colors. The side view in the right panel is rendered by rotating along the x-axis of the top view (the left panel) by 90° and then along the y-axis by 45° for a better view of the binding sites. Some ligands can have multiple binding poses within the same pocket; only one configuration is shown for clarity. Binding sites for ions or ion blockers are not shown.

**FIGURE 2:**
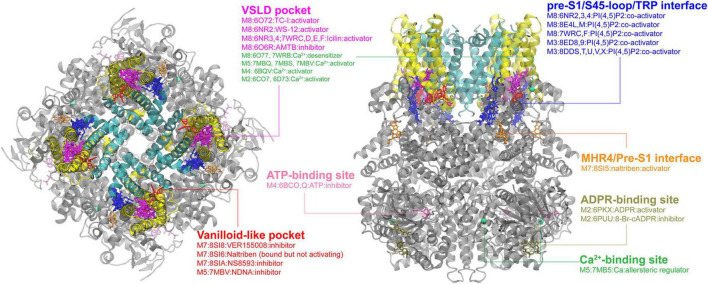
Ligand binding sites in TRPM channels. The TRPM8 structure with TC-I 2014-bound (PDB: 6O72) is used for visualization, with the pore region, the VSLD region and cytosolic domain colored in cyan, yellow, and gray, respectively. The major binding pockets or interfaces are highlighted using different colors. The top and side views are rendered in the same way as in the TRPV channel (see Figure 1).

**FIGURE 3:**
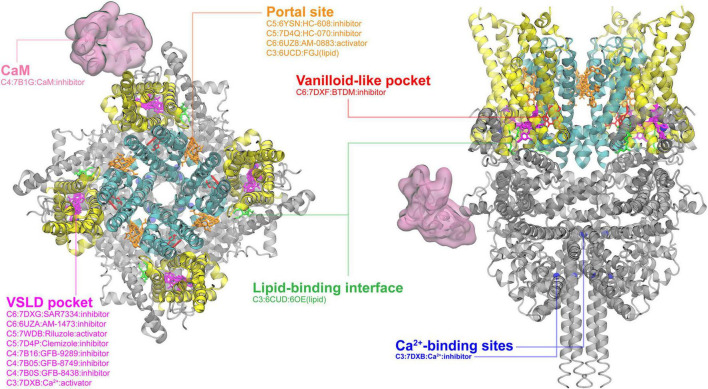
Ligand binding sites in TRPC channels. The SAR7334-bound TRPC structure (PDB: 7DXG) is used for visualization, with the pore region, the VSLD region and cytosolic domain colored in cyan, yellow, and gray, respectively. The major binding pockets or interfaces are highlighted using different colors. The Ca^2+^ binding sites (blue) are also included because Ca^2+^ plays a regulation role in the TRPC3 activity. The top and side views are rendered in the same way as in the TRPV channel (see Figure 1).

**FIGURE 4:**
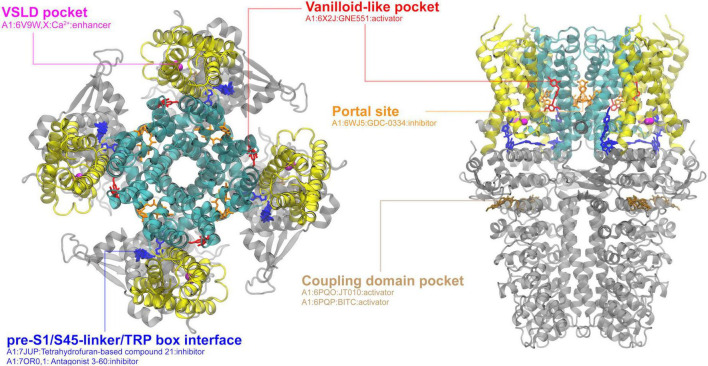
Ligand binding sites in the TRPA1 channel. The structure in complex with covalent agonist JT010 (PDB: 6PQO) is used for visualization, with the pore region, the VSLD region and cytosolic domain colored in cyan, yellow, and gray, respectively. The major binding pockets or interfaces are highlighted using different colors. The top and side views are rendered in the same way as in the TRPV channel (see Figure 1).

The TRP channel superfamily can be divided into two subgroups based on their structural features as well as cellular distributions (Montell, 2005). The first subgroup consists of TRPCs, TRPVs, TRPMs, and TRPA. They mainly distribute in the plasma membrane and share similar structural features. Structurally, this subgroup of TRP channels exists as tetramers, featuring six transmembrane (TM) helices in each protomer. Following the S6 helix, a TRP helix or so-called TRP box runs parallel to the membrane surface and is believed to play an important role in gating of TRP channels. The second subgroup includes TRPML and TRPP, which are located in the endosome membrane and do not have the TRP box. While TRP channels in the first subgroup contain large cytosolic domains from each protomer assembling as a skirt-like or multiple-layered structure enveloping a large cytosolic cavity (e.g., Figures 1–3), TRP channels from the second subgroup have large “extracellular” segments inserted between S1 and S2 and form a “cap”-like domain (Figure 4). In this review, we will focus on the ligand binding pockets shared among TRP channels and analyze the degree of binding site similarity and conservation among each subfamily, to provide some guidance for future TRP drug discovery and pharmaceutical research.

### TRPVs

As the most intensively investigated TRP subfamily, structures are available in different functional or ligand-bound states for all TRPV members. A thorough review of the ligand binding pockets in TRPV channels has been recently published, which summarizes 16 distinct binding sites in TRPV channels (Yelshanskaya and Sobolevsky, 2022). Though several new ligand-bound structures have been deposited in the PDB since, no additional binding site has been discovered. Here we will briefly summarize the most important sites (Figure 1), and discuss some of the very recent studies not included in the 2022 review. The so-called vanilloid site is the most frequently observed one. It is an interface cavity formed between S3, S4, S4-5 linker and the neighboring S5 and S6 helices. Ligands that bind to the vanilloid site include both activators, such as capsaicin (Cao et al., 2013; Kwon et al., 2021; Nadezhdin et al., 2021a), resiniferatoxin (Nadezhdin et al., 2021a; Zhang et al., 2021; Kwon et al., 2022), and inhibitors, such as capsazepine and SB-366791 in TRPV1 (Gao L. et al., 2016; Neuberger et al., 2023), econazole in TRPV5 and (4-phenylcyclohexyl)piperazine derivatives (PCHPDs) in TRPV6 (Hughes et al., 2018; Neuberger et al., 2021). It is worth mentioning that, when a ligand molecule is not present, the vanilloid site is generally occupied by lipid molecules, such as phosphatidylinositol lipid (PI) observed for TRPV1 (Gao L. et al., 2016; Zhang et al., 2021) and phosphatidylcholine lipid (PC) for TRPV3 (Nadezhdin et al., 2021b). The effects of lipid binding in the vanilloid site can be either inhibitory or excitatory and appear to have different physiological and functional implications among TRPV members (Cheng et al., 2022). Su et al. (2023) recently showed that binding of endogenous cholesterol to the vanilloid pocket inhibited the TRPV2 channel activity.

The second important binding site in TRPV channels is the S1-S4 bundle site or the VSLD pocket, formed by the S1-S4 helix bundle and the TRP helix. Several chemicals showing either activation (2-APB in TRPV3) or inhibition (2-APB-Br, Osthole in TRPV3 and ZINC17988990 in TRPV5) have been discovered to bind in this VSLD cavity (Figure 1; Supplementary Appendix Table 1). Recently, the agonists (4-alpha-PDD and GSK1016790A) and antagonists (HC-067047 and GSK2798745) bound structures of human TRPV4 were resolved, showing that both agonists and antagonists can bind to the VSLD cavity (Kwon et al., 2023; Nadezhdin et al., 2023b). The cryo-EM structures of TRPV channels also show lipids can occupy the VSLD cavity in absence of other ligands, the native functional implications of which need to be further investigated (Supplementary Appendix Table 1). The third major binding site is the portal site, which is the pocket formed by the S5 and the pore helix (PH) of one subunit plus the neighboring S6 helix. Cannabidiol or cannabidiol derivatives have been shown to bind to this portal site in TRPV2 (Gochman et al., 2023). Other compounds such as ZINC17988990 or ZINC9155420 inhibit TRPV5 by binding to this site (Hughes et al., 2019). Recently, the anesthetic dyclonine was also found to bind to the portal site in TRPV3, providing the structural basis of how this compound can relieve pain and itch in the traditionally topical applications (Neuberger et al., 2022). Some other binding sites (Figure 1) have also been found within TRPV members, such as central pore sites (sites along the central permeation pathway), the S4-5 site (the interface between VSLD and S5-6 pore helices, also referred as “deep” or “shallow” S4-5 in the 2022 review), the S2-3 site [the PI(4,5)P2 binding site in TRPV5], and the cytosolic calmodulin (CaM) binding site (Yelshanskaya and Sobolevsky, 2022). Those additional sites indicate the TRPV channels have the potential to be targeted by drugs in other less common but important interfaces or pockets. It is noteworthy that ligand binding to the same site can have different or sometimes completely opposite effects on the channel function, suggesting a high level of adaptability of the binding pockets and the likely presence of multiple coupling pathways and/or regulatory mechanisms with the TRP channel proteins.

### TRPMs

The TRPM subfamily members have also attracted intensive attention in recent years due to their important roles in sensing temperature, taste, oxidative state and osmolarity, cellular proliferation, cell death, neurological diseases and cancer progression (Jimenez et al., 2020). Cryo-EM structures have been determined for all TRPM channels in both the apo and bound states with different ligands, except for TRPM1 and TRPM6 (Huang et al., 2020). TRPM and TRPV channels share the similar architecture in the TM region (Figures 1, 2). Two of the major ligand binding pockets identified for TRPV channels, the vanilloid-like pocket and the VSLD pocket, are also present in TRPM channels (Figure 2). For the vanilloid-like pocket, inhibitor-bound structures such as VER155008 and NS8593 in TRPM7, N′-(3,4-dimethoxybenzylidene)-2-(naphthalen-1-yl)acetohydrazide (NDNA) in TRPM5, and activator-bound structure, Naltriben in TRPM7, have been reported (Ruan et al., 2021; Nadezhdin et al., 2023a), showing again the adaptability of this pocket. The VSLD cavity, so far only observed in the TRPM8 cryo-EM structures, can also accommodate ligands with either inhibitory (AMTB) or excitatory (TC-1, WS-12, icilin) effects. Interestingly, the portal site in TRPMs has not been found to bind any inhibitors or activators but can be occupied by lipids (Diver et al., 2019). Together with the large accessible groove on the inter-protomer surface, the portal site clearly has the potential to bind ligands and modulate the gating/activation process of the TRPM channels.

Another interesting feature of TRPMs is that the cytosolic domain of TRPMs usually contains of 4 melastatin homology regions (MHR1-4) instead of ankyrin repeats in TRPV channels. This variance provides several unique binding sites in TRPMs. The first one is the pre-S1/S4-5 loop/TRP helix interface, which provides a positively charged electrostatic environment to bind the PI(4,5)P2 molecule. So far, fourteen PI(4,5)P2-bound structures (seven for each TRPM3 and 8) reveal that the PI(4,5)P2 head group all binds into a similar position on this interface. However, the PI(4,5)P2 binding site might not be conserved in other TRPM channels, because the interacting residues are not conserved among TRPMs (Yin et al., 2019a). It would be interesting to dissect the PI(4,5)P2 binding in TRPMs because the important regulatory effect of PI(4,5)P2 on TRPMs has been long recognized (Runnels et al., 2002; Liu and Liman, 2003; Zhang et al., 2005; Nilius et al., 2006; Xie et al., 2011). Other less common cytosolic binding sites have also been reported individually in several TRPMs. The naltriben-bound TRPM7 structure reveals that the ligand binds to MHR4/pre-S1 interface and activates the channel by pulling MHR4 to the neighboring MHR repeat and triggering a rigid body rotation of the whole N-terminal domain (Nadezhdin et al., 2023a). In TRPM2, there is a unique ADPR (ADP ribose)-binding site, which is located at the cleft of the MHR1/2 and is far away from the central pore domain (Yin et al., 2019b). It has been shown that binding of ADPR to this site can activate the channel, while binding of its derivative 8-Br-cADPR can block the allosteric coupling from MHR3-4 to the central pore and lock the conformation in apo state (Huang et al., 2019; Yin et al., 2019b). In TRPM4, a nucleotide-binding site in the N-terminal region was found to bind to ATP, allosterically inhibiting the channel activity (Guo et al., 2017). These cytosolic sites are in general far away from the pore domain and thus pose an intriguing question on what long range coupling mechanism(s) would allow the ligand binding to control the pore domain.

### TRPCs

All members of the “canonical” TRP channels, TRPCs, were discovered in the late 1990s (Wes et al., 1995; Zhu et al., 1995, 1996; Okada et al., 1999). Their general structural features, functions and regulation have been reviewed elsewhere (Wang H. et al., 2020). Though not as extensively studied as the TRPV and TRPM subfamilies, over 30 structures of TRPCs have been determined, covering TRPC3-6 members (Supplementary Appendix Table 1). As shown in Figure 3, ligands in the bound structures of TRPCs mainly cluster into two common binding sites, namely, the portal and VSLD binding sites, as seen in TRPVs and TRPMs (Figures 1, 2). For TRPC4-6, there have been more extensive studies attempting to determine the ligand-bound structures, including both activators and inhibitors. Only one structure of the agonist (AM-0883)-bound human TRPC6 was captured a more open state among all reported TRPC structures. The binding of the AM-0883 in the portal site, situated between the S6 of one monomer and the pore helix (PH) of another, tilts S6 as well as the VSLD and S4-S5 linker, suggesting a further rotation of S6 to release the hydrophobic seal in the open state pore of TRPC6 (Bai et al., 2020). Interestingly, the portal site also accommodates inhibitors HC-070 and HC-608 in TRPC5 and lipids in TRPC3 (Wright et al., 2020; Song et al., 2021). A second highly populated binding site among TRPC4-6 is the VSLD pocket. There are a handful of inhibitors and an activator that bind to this pocket (Bai et al., 2020; Vinayagam et al., 2020; Song et al., 2021; Guo et al., 2022; Yang et al., 2022; Figure 3). The activator/inhibitor pair riluzole and clemizole have been determined to bind to this pocket in TRPC5. These ligands are used pharmacologically to combat amyotrophic lateral sclerosis (ALS) and anxiety and depression, respectively (Song et al., 2021; Yang et al., 2022). The vanilloid-like pocket prominent in TRPV and TRPM channels has been observed to be occupied by [2-(1,3-benzodioxol-5-ylamino)-1,3-thiazol-4-yl]-[(3R,5S)-3,5-dimethylpiperidin-1-yl]methanone (BTDM) in TRPC6 (Figure 3; Guo et al., 2022). TRPC3 has only been resolved in the closed state(s) with observed binding by unidentified lipids in a binding pocket between S1 and the pre-S1 elbow (Fan et al., 2018) and by Ca^2+^ in several intracellular regions (Guo et al., 2022; Figure 3).

### TRPA1

TRPA1 is the sole member of the TRPA subfamily, characterized by its 16 ankyrin repeats (the longest among TRP channels) and a TM region is structurally very similar to TRPVs (Paulsen et al., 2015). Due to its previously observed pain- and irritant-sensitivity, it has been studied extensively with various ligands (Meents et al., 2019). TRPA1 structure usually could be divided into three layers with the top, middle and bottom layers consisting of the TM domain, the coupling domain and the ankyrin repeat domain, respectively. As shown in Figure 4, all cryo-EM-resolved ligand-bound structures together show the four familiar binding sites as already discussed above: the vanilloid-like site, the VSLD pocket, the portal site and the pre-S1/S4-5 loop/TRP helix interface, in addition to a unique coupling domain pocket. The portal site, formed by S5/S6/PH, can bind with the GDC-0334 inhibitor, which reduces airway inflammation as asthma treatment (Balestrini et al., 2021). Interestingly, a separate study of compound-21 (C21) has also shown to reduce airway inflammation, though by binding to the pre-S1/S4-5 linker/TRP box interface (Terrett et al., 2021), which is often occupied by regulatory lipids in TRPM and TRPV channels. GNE551, an agonist of TRPA1, binds in the vanilloid-like pocket, though still resulting in a non-conductive state of TRPA1 (Liu et al., 2021). The most unique binding site of TRPA1 is the coupling domain pocket (Figure 4). The coupling domain pocket is believed to be important for the “electrophile sensing” of TRPA1 due to the presence of several cysteine residues (Bahia et al., 2016). TRPA1 is a great example of the structural similarities and differences among the TRP subfamilies that allow both conserved and unique binding pockets.

### TRPMLs

**FIGURE 5:**
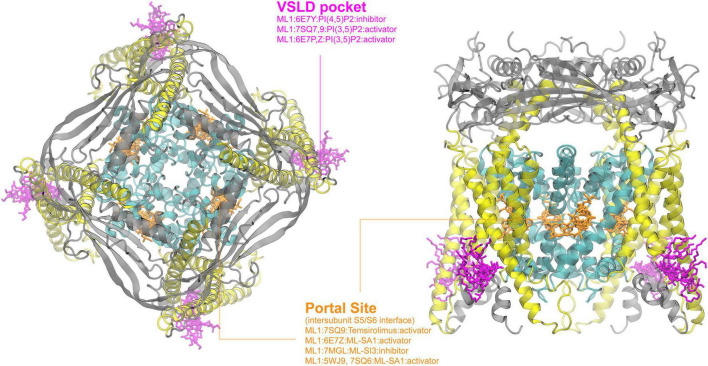
Ligand binding sites of TRPML channels. The PI(4,5)P_2_-bound TRPML1 structure (PDB: 6E7Y) is used for visualization, with the pore region, the VSLD region and extracellular domains colored in cyan, yellow, and gray, respectively. The top **(left)** and side **(right)** views are rendered as described in Figure 1. Existing complex structures of TRPML only show two major binding pockets.

Primarily localized in the endolysosomal membrane of mammalian cells, TRPML is one of the least studied TRP subfamilies (Zhang X. et al., 2018). Cryo-EM structures of all members of the TRPML family have been resolved, but only TRPML1 has solved structures in both open and closed states with various lipids and ligands present. As a homotetrameric Ca^2+^-permeable, nonselective, cation channel, TRPML1 regulates lysosomal calcium signaling, lipid trafficking, and autophagy-related processes. As such, loss-of-function mutation of TRPML1 is associated with a neurodegenerative disorder, known as Mucolipidosis type IV (MLIV) (Schmiege et al., 2021). As shown in Figure 5, current cryo-EM structures enriched all ligands or internal mediators (such as PIP2 molecules) into two major binding pockets: the VSLD pocket and the portal site. Lipids PI(3,5)P_2_ and PI(4,5)P_2_ both have been resolved to bind in the VSLD pocket, where polar and hydrophilic residues are distributed similar to the corresponding pocket in other TRP channels (see above). Interestingly, PI(3,5)P_2_ has been observed to promote channel opening, while PI(4,5)P_2_ can act as channel suppressor (Fine et al., 2018). It was proposed that due to the different phosphate group locations, R403 and Y355 are positioned to promote pi-cation interactions in PI(3,5)P_2_ but not PI(4,5)P_2_, affecting the movement of the S4-5 linker and further facilitating pore opening (Fine et al., 2018). The portal site in TRPML1, which is similar to the one in TRPV channels, is formed by the TM interface between S5 of one domain and the neighboring S6. It hosts both the agonist ML-SA1 and antagonists ML-SI3 and temsirolimus through mostly hydrophobic interactions (Fine et al., 2018; Schmiege et al., 2021). ML-SA1 was observed, unlike ML-SI3, to promote pi-pi interactions in the portal site, pulling S6 away from the central axis and thus opening the pore (Fine et al., 2018). A similar open state was observed with temsirolimus binding in tandem with PI(3,5)P_2_ as was observed with ML-SA1 alone (Gan et al., 2022). Interestingly, the VSLD pocket and the portal site in TRPML1 have been shown to both independently and synergistically modulate the channel, with PI(3,5)P_2_ binding observed even in a ML-SI3 bound state (Schmiege et al., 2021; Gan et al., 2022).

### TRPPs

As the most primitive and ancient member of the TRP family, TRPP family was found to present in both animals and yeast, and mutations implicate the autosomal dominant polycystic kidney disease (ADPKD) (Gees et al., 2012; Samanta et al., 2018). TRPP2 (polycystin-2, polycystin kidney disease-2 or PKD2) and TRPP3 (polycystin-2 like, or PKD2L1) are Ca^2+^-activated cation channels which are structural homologous to other TRP ion channels in terms of the six transmembrane helices. Structural studies have revealed cryo-EM structures of TRPP2 and TRPP3 either in the apo state or in the PIP2-bound state (Supplementary Appendix Table 1). Unfortunately, the PI(4,5)P2 and PI(3,5)P2 were not well-resolved based on cryo-EM density maps of TRPP2 (Wang Q. et al., 2020). Despite this ambiguity, the authors found the density of those PIP2 molecules to be located at the vanilloid-like pocket (Wang Q. et al., 2020). The structural information is still very limited to have a better understanding of ligand binding in TRPP ion channels and thus requires further investigation, though the conserved six transmembrane helices structure suggests that the common binding sites comprised of elements from the TM region discussed in all the above families might be also very likely the binding sites in TRPP channels.

## Molecular mechanisms of TRP activation and regulation

The remarkable sensory roles of TRP channels are conferred by the complex dynamic properties of the protein conformations and how they can be delicately controlled by various physical and chemical stimuli. A deep understanding of the molecular mechanisms of these controls is critical for successful rational approaches targeting TRP channels. Activation of an ion channel can be divided into three general steps. First, the “sensor” domain or element needs to respond to the given stimuli, which typically involve certain local conformational changes and movements. Second, the conformational response of the sensor, which is typically distal from the ion-conducting pore, needs to be transduced to the pore domain through intramolecular coupling pathway(s). Lastly, the pore needs to undergo to an opening transition and release the gating element for ion permeation. A drug molecule could interfere with any or all of these three steps of channel activation. The abundant structural data on TRP channels have provided a solid basis for understanding the inner work of these channels. However, a recent global analysis of TRP channel TM domain structures revealed that most available structures represent non-conducting states, leaving much to be learned about the gating transitions alone (Huffer et al., 2020). Even more questions remain to be answered regarding the identities and movements of the sensors as well as the sensor-pore coupling. Below, we summarize the current understanding of the three general steps in TRP channel activation.

### Sensor elements in the TRP channels

The sensory roles of TRP channels are well-documented in term of somatosensation. TRPV1-4, TRPM2, TRPM3 and TRPM5 have been reported to be heat sensing, whereas TRPC5, TRPM8 and TRPA1 can be cold sensing (Talavera et al., 2005; Wetsel, 2011; Voets, 2014; Wang and Siemens, 2015; Kashio and Tominaga, 2022). TRPVs and TRPA1 channels can sense touch, pain and itch (Mickle et al., 2015; Sun and Dong, 2016; Moore et al., 2018). In addition, TRPV4 has been shown to be capable of mechanosensation, including osmo-sensation (Christensen and Corey, 2007). Sensation of the redox status has also been reported for TRPC5, TRPV1 and TRPA1 (Takahashi and Mori, 2011; Ogawa et al., 2016). In addition to the physical stimuli, many chemicals, synthetic and natural, can regulate TRP channel functions, as extensively discussed above.

#### Thermo-sensor

Extensive efforts have been dedicated to pinpoint the thermo-sensor elements of the thermo-TRPs. Many candidates have been evaluated through deletion, mutagenesis and chimeragenesis, so far without reaching a conclusive identification (Diaz-Franulic et al., 2021; Luu et al., 2023). Temperature sensing elements have been proposed throughout the channel structure including: the ankyrin repeat domain for TRPA1 (Cordero-Morales et al., 2011) and TRPV1 (Saito et al., 2016; Ladrón-de-Guevara et al., 2020; Hori et al., 2023), a membrane proximal domain (the N-terminal region connects ankyrin repeats to the S1 helix) for TRPV1-V3 (Yao et al., 2011; He et al., 2017; Liu and Qin, 2021), the whole VSLD for TRPV1 (Kim et al., 2020), the pore turret (Yang et al., 2010; Cui et al., 2012; Du et al., 2020) [although contradicting with a study showing the torrent-deleted TRPV1 remains thermosensitivity (Liao et al., 2013)], the pore helix domain for TRPV1 (Myers et al., 2008) and TRPA1 (Wang et al., 2013), a loop after the pore helix plus the S6 helix for TRPV3 (Grandl et al., 2008), the outer pore loop region for TRPV1 (Grandl et al., 2010), the whole pore domain (S5-S6) for TRPV1 (Zhang F. et al., 2018) and the C-termini for TRPV1 (Vlachová et al., 2003; Brauchi et al., 2006; Joseph et al., 2013). These studies have been plagued by the different experimental conditions/procedures being employed and ambiguity in interpretation. It is possible that there is no single thermo-sensing element in a given TRP channel; instead, the temperature driven conformational transition may emerge from the cooperative property of the entire oligomer assembly within its native membrane environment.

On the other hand, it was also proposed that the thermosensitivity of the thermoTRP channels may not be necessarily attributed to a specific sensor element or domain (Clapham and Miller, 2011; Yeh et al., 2023). Instead, thermoTRP channel activation may be accompanied by large molar heat capacity differences, such that both the activation enthalpy and entropy would be both temperature dependent and the temperature dependence of the open-close equilibrium would be always non-monotonic. Such a model could give rise to both cold and hot activation behaviors, depending on temperature where the open-close equilibrium constant minimizes. It was further proposed that a major contribution to the molar heat capacity is solvation or desolvation of hydrophobic residues and charged ones during activation, which could be delocalized throughout the whole channel protein. This model has been successfully applied to rationally engineer a canonical voltage-sensing potassium channel to confer temperature sensitivity, by varying the polarity of residues in the VSD that undergoes state-dependent changes in solvation (Chowdhury et al., 2014). The molecular basis for the successful design was further confirmed by NMR and molecular dynamics simulations, which reveal increased hydration in the VSD of the engineered channel at high temperatures (Chen et al., 2021).

It is worthy of noticing that cryo-EM studies of TRPV3 have revealed the closed, the heat-induced sensitized state as well as the open state, providing a structural foundation for understanding the molecular mechanism of temperature-sensing (Singh et al., 2019; Nadezhdin et al., 2021b). Comparison of those different states revealed a mutually dependent conformational wave, which involved secondary structural rearrangements of the S2-3 linker and N-/C-termini, and rigid body translational movements involving ARD, the S4-5 linker and pore domain helices (Nadezhdin et al., 2021b). It was thus proposed that the distributed multi-domain conformational wave could be triggered at any localized “sensor” within the wave itself (Nadezhdin et al., 2021b). Nevertheless, heat-induced secondary structure rearrangements could shape the energetics of close-to-open equilibrium. For example, exposure of 10∼15 hydrophobic residues per subunit, to give a ΔH value as large as ∼90 kcal/mol (Nadezhdin et al., 2021b). Nonetheless, it remains challenging to dissect the contributions of individual regions involved in the conformational wave and to explore whether the heat capacity differences in thermoTRP channels can be attributed to localized thermo-sensors or it arises from delocalized contributions throughout the channel.

#### pH-sensor

pH, as one important aspect of the physiological conditions for living cells, has shown to regulate many TRP channels. TRPV1 was the first TRP channel found to be potentiated and even directly activated by extracellular acidification (Caterina et al., 1997; Tominaga et al., 1998; Baumann and Martenson, 2000). Two extracellular Glu residues (E600 and E648, located at the pore turret and the pre-S6 loop, respectively) were proposed to be the pH-sensing elements of the pH-induced potentiation and activation in TRPV1 (Jordt et al., 2000). Another study suggests that F660 located in the pore domain is the key pH-sensor of TRPV1 (Aneiros et al., 2011). Extracellular protons have also been shown to activate TRPV4 (Suzuki et al., 2003), and potentiate TRPA1 (Takahashi et al., 2008; de la Roche et al., 2013), TRPM6 (Li et al., 2007), TRPM7 (Jiang et al., 2005; Li et al., 2007; Numata and Okada, 2008), TRPC4 (Semtner et al., 2007), TRPC5 (Semtner et al., 2007; Kim et al., 2008) and TRPP3 (Inada et al., 2008). Negatively charged residues (Asp or Glu) in the pore turret, the pre-S6 loop as well as the selectivity filter have been frequently proposed to be the pH-sensing elements in these channels (Zheng, 2013), although specific locations of those charge residues may differ greatly among those members. Extracellular protons could also be inhibitory for some TRP channels, including TRPV3 (Wang et al., 2021), TRPV5 (Yeh et al., 2003), TRPM2 (Du et al., 2009), TRPM5 (Liu et al., 2005), and TRPC6 (Semtner et al., 2007).

Furthermore, intracellular alkalization can activate TRPV1 with H378 in the ARD proposed to be the sensor (Dhaka et al., 2009). Intracellular proton-induced potentiation and activation was also reported in TRPV3 involving a N-terminal H426 (Cao et al., 2012) or the S2-3 linker (Gao L. et al., 2016), whereas intracellular proton-induced inhibition has been observed for TRPV5 involving a proximal C-terminal K607 (Yeh et al., 2005) and TRPM2 involving the S4-5 linker (Du et al., 2009).

Although there is some consensus in terms of pH-sensors in individual TRP members, the proposed key residues are often scattered in both the extracellular and intracellular domains. The implication is that TRP channels do not share conserved pH-sensing elements or mechanism. More studies are required to elucidate the molecular mechanisms of proton regulation in TRP channels.

#### Mechanosensor

The nature and location of the mechanosensor domain in mechanosensitive TRP channels (for example, TRPV4, TRPA1, TRPC1, TRPC6, and TRPP2) also remain largely elusive (Lin and Corey, 2005; O’Neil and Heller, 2005). Several proposals have been discussed. For example, the ankyrin repeats domain in the TRP channels was proposed to function like a molecular “spring” during the mechanical force-induced gating (Corey et al., 2004; Howard and Bechstedt, 2004). Further, it has been debated whether a TRP channel is directly transducing mechanical signals or it is indirectly regulated by being a downstream receptor of the signaling pathway (Christensen and Corey, 2007). Evidence has suggested that epoxyeicosatrienoic acids, a type of cellular secondary messenger, can directly activate TRPV4 (Vriens et al., 2005). Very recently, two independent cryo-EM studies captured the human TRPV4-RhoA (a small GTPase) complexes showing RhoA interacts extensively with the ARD domain (Kwon et al., 2023; Nadezhdin et al., 2023b). Given that RhoA is a membrane-anchoring protein, it is possible that RhoA plays a role in connecting or transducing membrane surface or morphological changes to the TRPV4 channel (Kwon et al., 2023; Nadezhdin et al., 2023b). More intensive studies are required to dissect the role of mechanosensitive TRP channels in mechanical transduction and the possible existence of mechanosensor domains.

#### Chemosensor

As a sole member in the TRPA subfamily, TRPA1 has long been recognized as the “chemonociceptor” due to its ability to “sense” a wide range of noxious chemical compounds or environmental irritants (Meents et al., 2019; Manolache et al., 2021). Mechanistically, TRPA1 can be activated by thio-reacting electrophile irritants using an array of cysteine residues loaded at the N-terminal domain (Hinman et al., 2006; Macpherson et al., 2007; Bahia et al., 2016), and also binds non-covalently with other non-reactive chemicals in a way of using the traditional binding pocket(s) (Xu et al., 2006; Karashima et al., 2007; Lee et al., 2008; Liu et al., 2021). The chemosensor of TRPA1 discussed here only refers to the covalent binding module. Several functional studies have consistently revealed that highly reactive C621 (human TRPA1 numbering, uniprot: O75762) plays a key role in covalent binding of electrophiles, though inconsistency exists with other potential cysteine sites based on mutagenesis data (Hinman et al., 2006; Macpherson et al., 2007; Bahia et al., 2016, p. 621). The role of C612 as the “chemosensor” element of TRPA1 has been further supported by cryo-EM structures showing agonist-modified C612 (Suo et al., 2020; Zhao J. et al., 2020).

### Mechanism of sensor-pore coupling during TRP channel activation

At present, there are relatively limited studies on the sensor-pore coupling mechanisms of the TRP channels, to a large degree due to much ambiguity in the sensor elements (see above). Existing analysis is largely based on structural data alone. Here, we highlight two conformational switches that have been the most extensively investigated.

#### C-terminal switch

An interesting structural rearrangement during the close/open transition found primarily in thermoTRPVs (TRPV1-V4) involves a loop-to-helix transition in the C-terminus, termed “C-terminal switch” (Zubcevic et al., 2019; Deng et al., 2020). TRPV channels share a C-terminal domain (CTD) following the TRP helix, which coils back to the coupling domain (defined as the domain from right after ankyrin repeat 6 to the pre-S1 helix), forming an interacting network with the neighboring ankyrin repeat domain (Cao, 2020; Pumroy et al., 2020). The CTD was proposed to be involved in temperature-induced gating in several cases (Vlachová et al., 2003; Brauchi et al., 2004, 2006, 2007). The C-terminal switch was discovered in the structural studies of a sensitized phenotype (the K169A mutant) in the human TRPV3 that breaks an important salt bridge between CTD and the neighboring ARD (Zubcevic et al., 2019). The K169A mutation induces distal CTD to undergo coil-to-helix transition, altering the position of CTD and the interactions at the inter-protomer interface. It was thus proposed that the C-terminal loop-to-helix transition represents a functional “switch” during TRPV channel gating (Zubcevic et al., 2019). Similar helical CTD was also observed in the rat TRPV2 for both the apo and the agonist-bound (cannabidiol) structures (Pumroy et al., 2019). Besides, in the recently resolved TRPV4-RhoA complex, binding of 4-αPDD also triggers the C-terminus transit from a loop to a α-helix in the captured open state (Nadezhdin et al., 2023b). In another study of mouse TRPV3, although the distal CTD was assigned as a loop in the open state, it was proposed to be a “latch” that needs to be released and unwrapped from the N-terminal beta-sheet to sensitize the channel (Singh et al., 2019). A recent cryo-EM structure of squirrel TRPV1 also revealed that the C-terminus “hook” wraps around the interdomain N-terminal beta-sheet (Nadezhdin et al., 2021a). In many other cases, the CTD was not well-resolved in thermoTRPV channels, due to either limitation of the cryo-EM resolution or the inherent structural flexibility. Nonetheless, it has been suggested that the CTD conformational transition plays in the temperature-induced gating of TRP channels.

#### Coupling of the S6 and TRP helices

Changes in the S6 and TRP helix coupling have also been observed in the ligand-induced open/close transitions of the TRP channels. This type of transition usually involves elongation of the S6 helix and/or shortening of the TRP helix. It was firstly discovered in the captured open state of TRPV3 where the S6 helix is elongated by two helical turns compared with the closed state (Singh et al., 2018). A similar pattern was discovered later in ligand-induced activation of the human TRPV3 (Zubcevic et al., 2019), temperature-induced opening of the mouse TRPV3 (Singh et al., 2019) and more recently in the gain-of-function mutation-induced opening of the mouse TRPM7 (Nadezhdin et al., 2023a). Besides, structural determination of PIP2-bound TRPV5 revealed that PIP2 binding induces lengthening of the S6 helix for about 1 helical turn and shortening of the TRP helix in order to form favorable salt bridge interactions with PIP2 head group (Hughes et al., 2018). Changes on the helix rearrangement between the S6 and TRP helix will likely alter the pore-sensor coupling upon stimulation of the channel, which often swivels or “pulls” the S6 helix outward to open the central pore (Hughes et al., 2018; Zubcevic et al., 2019; Nadezhdin et al., 2023a). It is worth noting that the C-terminal switch and coupling of S6-TRP helices are not mutually exclusive during the close/open transitions (Hughes et al., 2018; Zubcevic et al., 2019).

### How does the pore open: gating of TRP channels

Generally speaking, TRP channels can have two constriction regions that may serve as the gate – the selectivity filter region and the bundle-crossing formed by the pore-lining S6 helices. The existence of a selectivity filter gate varies among subfamilies. The selectivity filter of TRPV1-3 allows ions to enter the pore even in the inactive state, indicating that the selectivity filter might not be a gate (Jara-Oseguera et al., 2019). Global structure alignments showed that the selectivity filter regions of nonselective TRP channels have large variations on their radii of opening, indicative of significant intrinsic flexibility (Huffer et al., 2020). Selective TRP members, such as TRPV5-6 and TRPM4-5, tend to contain narrower filter regions compared with nonselective ones (Huffer et al., 2020). Further, it has been shown that the cytosolic S6 activation gate formed by bundle-crossing (also being called “hydrophobic seal”) is a consistent feature among most all TRP channels (Cao, 2020; Huffer et al., 2020; Pumroy et al., 2020). Additional contribution to TRP channel gating may come from the hydrophobic inner pore region between the bundle-crossing and selectivity filter, which may undergo spontaneous dewetting transitions and form a vapor barrier to block ion permeation (Huang and Chen, 2023). An important caveat is that most currently resolved TRP structures, either with ligand bound or not, have a rather narrow cytosolic gate and thus represent a closed pore (Huffer et al., 2020), which present extra challenges in delineating the gating mechanisms.

One of the more notable and general structural elements in the gating of TRP channels is probably the π-helix in a single turn of the S6 helix. Since many TRP channels form a “bundle-crossing” lower gate, it was proposed that the flexibility of the π-helical turn in the middle of the S6 helix allows bending of the S6 helix and thus may enable channel opening. The π-helices resemble the glycine hinge or the proline hinge discovered previously in potassium channels. As the TM regions of TRP channels are highly conserved, almost all TRP subfamilies can find cases exhibiting state-dependent π-helices in their captured structures in either closed, sensitized or open states (Zubcevic and Lee, 2019). TRPV6 is an exemplary case where the α-to-π transition at an alanine hinge of the S6 helix dictates the close-to-open conformational transition (McGoldrick et al., 2018). The π-helix can induce bending and rotation of S6, changing the pore-lining residues to create a more ion-favorable hydrophilic environment (McGoldrick et al., 2018). In TRPV3, the α-to-π transition occurs during ligand-induced sensitization, which widens the pore slightly and exposes different groups of pore-lining residues (Singh et al., 2018; Zubcevic et al., 2018). Studies have also found some TRP members can also have π-to-α transition going from closed state to sensitized or open state, such as the most recently reported TRPV4 (Nadezhdin et al., 2023b), whereas some TRP channels exhibit π-helix in the S6 helix in both the closed and open state, including the most recent case of TRPV3 and TRPM7 (Zubcevic and Lee, 2019; Nadezhdin et al., 2021b,2023a). It is worth noting that a conserved feature among all TRP channels is that the pore-lining residues in the inner pore region of S6 in the closed state are usually hydrophobic ones, forming a so-called “hydrophobic seal.” For example, the lower portion of S6 contains a highly conserved sequence, LLLNMLI, among TRPV1-4. The hydrophobic lower pore region present in the deactivated state suggest a general role of hydrophobic gating mechanism among TRP channels (Aryal et al., 2015; Yazdani et al., 2020). The dewetting transition involved in hydrophobic gating can be readily controlled by α-to-π or π-to-α transitions modifying the pore geometry and surface hydrophobicity.

## Concluding discussion

As multifunctional proteins intimately involved in diverse physiological processes, TRP channels are considered exciting and potentially rewarding therapeutic targets. Many modulators of this ion channel family are under development, and several have reached clinical trials (Moran et al., 2011; Moran and Szallasi, 2018; Iftinca et al., 2021; Koivisto et al., 2022). At the same time, TRP channels have presented critical challenges due to their complex, polymodal activation and regulation and complex roles in physiological functions, which frequently leads to potential issues with clinical efficacy, safety and side effects. Overcoming these issues has been plagued by important gaps in the current understanding of TRP channel function at the molecular level. While hundreds of structures of are now available for TRP channels in both apo and bound states, they alone do not readily reveal functional mechanisms. For example, only a handful of the “open” structures represent truly conductive states (Huffer et al., 2020). Many agonists and antagonists can bind to similar pockets, without leading to apparent conformational changes in the channel protein. We still do not have a concrete understanding of how the TRP channels may sense temperature, mechanical force, osmotic pressure or voltage. These critical gaps in the fundamental understanding of the TRP channel function make it extremely challenging for any rational attempt to optimize lead chemical matters or discover novel ones.

Notwithstanding many important challenges, intensive research into the molecular basis of the TRP channel function has generated a rich set of structural and functional data. As summarized in this review, high resolution structures are now available for all subfamilies of TRP channels, often times in multiple functional states and/or several ligand-bound states. Some mechanistic features of TRP channel gating are also emerging, such as the α-to-π transition of pore-lining S6 helix and the potential role of hydrophobic gating. These structures together reveal major binding pockets present in the TRP channels. Multiple binding sites, inside and outside of the membrane bilayer, have been identified for some members of this family with some pockets seemingly more druggable than others. In some TRP channels, the only identified pockets reside deep in the membrane, which can lead to challenges in identifying development candidates with drug-like physicochemical properties. Low solubility and permeability may result in poor bioavailability, limiting the effectiveness of the drug. There is an urgent need and exciting opportunity to leverage this rich set of structural and functional data to further elucidate the three general steps of channel activation and regulation, namely, sensor movements, sensor-pore coupling, and pore opening transitions. This will require concerted efforts from computation, structural biology, medicinal chemistry, electrophysiology, and pharmacology. An ever improving understanding the channels’ activation and regulatory mechanisms will guide the drug design efforts and open new possibilities and venues for targeting the TRP channels in therapeutics.

## Author contributions

JH: Writing—review and editing, Data curation, Visualization, Writing—original draft. AK: Data curation, Visualization, Writing—original draft. MY: Conceptualization, Writing—original draft, Writing—review and editing. JC: Conceptualization, Funding acquisition, Writing—review and editing.
