# Supplementary material for: Targeting TRP channels: recent advances in structure, ligand binding, and molecular mechanisms
Source: Front Mol Neurosci. 2024 Jan 11;16:1334370. doi: 10.3389/fnmol.2023.1334370 (PMC10808746; doi:10.3389/fnmol.2023.1334370)
Supplement: Supplementary file 1 [file Table_1.docx]

### Targeting TRP Channels: Recent Advances in Structure, Ligand Binding and Molecular Mechanisms

**Supplementary Information**

Jian Huang^1^, Aron Korsunsky^1^, Mahdieh Yazdani^2*^, and Jianhan Chen^1*^

^1^Department of Chemistry; University of Massachusetts, Amherst, MA 01003, USA

^2^Modeling and Informatics, Merck & Co., Inc., West Point, Pennsylvania 19486, United Statesss

* Corresponding Authors: [mahdieh.yazdani@merck.com](mailto:mahdieh.yazdani@merck.com) (MY), [jianhanc@umass.edu](mailto:jianhanc@umass.edu) (JC)

####

#### Appendix Table1: TRP structures in PDB as of October 2023. The binding site column is left blank in cases where the ligand densities are not unambiguously resolved.

| **TRP** | **Species** | **Ligand** | **Binding site** | **PDB** | **Year** | **Reference** |
| --- | --- | --- | --- | --- | --- | --- |
| **TRPV6** | Human | Ruthenium red | Selectivity filter | 7S8B | 2021 | <https://doi.org/10.1038/s41467-021-26608-x> |
| **TRPV6** | Human | Phytoestrogen Genistein | center pore site | 8FOA | 2022 | <https://doi.org/10.1038/s41467-023-38352-5> |
| **TRPV6** | Rat | Gd^3+^ |  | 5WOA | 2017 | <https://doi.org/10.1038/s41598-017-10993-9> |
| **TRPV6** | Rat | Gd^3+^ |  | 5IWT | 2016 | <https://doi.org/10.1038/nature17975> |
| **TRPV6** | Human | Econazole | vanilloid pocket | 7S8C | 2021 | <https://doi.org/10.1038/s41467-021-26608-x> |
| **TRPV6** | Human | Calmodulin | intracellular | 6E2F | 2018 | <https://doi.org/10.1126/sciadv.aau6088> |
| **TRPV6** | Rat | Calmodulin | intracellular | 6E2G | 2018 | <https://doi.org/10.1126/sciadv.aau6088> |
| **TRPV6** | Rat | Ca^2+^ |  | 5WO9 | 2017 | <https://doi.org/10.1038/s41598-017-10993-9> |
| **TRPV6** | Rat | Ca^2+^ |  | 5IWP | 2016 | <https://doi.org/10.1038/nature17975> |
| **TRPV6** | Rat | Ba^2+^ |  | 5IWR | 2016 | <https://doi.org/10.1038/nature17975> |
| **TRPV6** | Human | 4-phenylcyclohexylpiperazine inhibitors 3OG, 30, 31 | Vanilloid Pocket and center pore site | 7K4D  7K4E  7K4F | 2020 | <https://doi.org/10.1126/sciadv.abe1508> |
| **TRPV6** | Rat | 4-phenylcyclohexylpiperazine inhibitor Br-cis-22a | Vanilloid Pocket and center pore site | 7D2K | 2020 | <https://doi.org/10.1126/sciadv.abe1508> |
| **TRPV6** | Human | --- |  | 7K4A | 2020 | <https://doi.org/10.1126/sciadv.abe1508> |
| **TRPV6** | Human | 4-phenylcyclohexylpiperazine inhibitor Br-cis-22a | Vanilloid Pocket and center pore site | 7K4B  7K4C | 2020 | <https://doi.org/10.1126/sciadv.abe1508> |
| **TRPV6** | Rat | 2-APB-Br | VSLD | 6D7V  6D7X | 2018 | <https://doi.org/10.1038/s41467-018-04828-y> |
| **TRPV6** | Rat | 2-APB | VSLD | 6D7O  6D7Q  6D7T | 2018 | <https://doi.org/10.1038/s41467-018-04828-y> |
| **TRPV6** | Human | tetrahydrocannabivarin | portal site | 8SP8 | 2023 | <https://doi.org/10.1038/s41467-023-40362-2> |
| **TRPV6** | Human | --- |  | 8FOB | 2022 | <https://doi.org/10.1038/s41467-023-38352-5> |
| **TRPV6** | Human | --- |  | 7S88  7S89 | 2021 | <https://doi.org/10.1038/s41467-021-26608-x> |
| **TRPV6** | Human | --- |  | 6BO8  6BO9  6BOA | 2018 | <https://doi.org/10.1038/nature25182> |
| **TRPV6** | Rat | --- |  | 6BOB | 2018 | <https://doi.org/10.1038/nature25182> |
| **TRPV6** | Rat | --- |  | 6D7P | 2018 | <https://doi.org/10.1038/s41467-018-04828-y> |
| **TRPV6** | Human | --- |  | 6D7S | 2018 | <https://doi.org/10.1038/s41467-018-04828-y> |
| **TRPV6** | Rat | --- |  | 5WO6  5WO7  5WO8 | 2017 | <https://doi.org/10.1038/s41598-017-10993-9> |
| **TRPV6** | Rat | --- |  | 5IWK | 2016 | <https://doi.org/10.1038/nature17975> |
| **TRPV5** | Rabbit | Zinc9155420 | portal site | 6PBF | 2019 | <https://doi.org/10.7554/eLife.49572> |
| **TRPV5** | Rabbit | Zinc17988990 | portal site | 6PBE | 2019 | <https://doi.org/10.7554/eLife.49572> |
| **TRPV5** | Rabbit | PI(4,5)P2 | S23 site | 7T6M  7T6Q | 2021 | <https://doi.org/10.1016/j.celrep.2022.110737> |
| **TRPV5** | Rabbit | Calmodulin | CaM site | 7T6R | 2021 | <https://doi.org/10.1016/j.celrep.2022.110737> |
| **TRPV5** | Rabbit | PI(4,5)P2 | S23 site | 6DMU | 2018 | <https://doi.org/10.1038/s41467-018-06753-6> |
| **TRPV5** | Rabbit | Lipid-bound |  | 6DMR | 2018 | <https://doi.org/10.1038/s41467-018-06753-6> |
| **TRPV5** | Rabbit | Calmodulin | CaM site | 6DMW | 2018 | <https://doi.org/10.1038/s41467-018-06753-6> |
| **TRPV5** | Rabbit | Econazole | Vanilloid pocket | 6B5V | 2018 | <https://doi.org/10.1038/s41594-017-0009-1> |
| **TRPV5** | Rabbit | Calmodulin | CaM site | 6O20 | 2019 | <https://doi.org/10.1073/pnas.1820323116> |
| **TRPV5** | Rabbit | --- |  | 6O1N  6O1P | 2019 | <https://doi.org/10.1073/pnas.1820323116> |
| **TRPV5** | Rabbit | --- |  | 7T6J  7T6K  7T6L  7T6N  7T6O | 2021 | <https://doi.org/10.1016/j.celrep.2022.110737> |
| **TRPV5** | Rabbit | --- |  | 7T6P | 2021 | <https://doi.org/10.1016/j.celrep.2022.110737> |
| **TRPV5** | Rabbit | --- |  | 6O1U | 2019 | <https://doi.org/10.1073/pnas.1820323116> |
| **TRPV4** | Human | --- |  | 8FC9 | 2023 | <https://www.nature.com/articles/s41467-023-39345-0#Sec21> |
| **TRPV4** | Human | GSK279 | VSLD | 8FC7 | 2023 | <https://www.nature.com/articles/s41467-023-39345-0#Sec21> |
| **TRPV4** | Human | GSK101 | VSLD | 8FCB 8FC8 | 2023 | <https://www.nature.com/articles/s41467-023-39345-0#Sec21> |
| **TRPV4** | Human | 4α-PDD | VSLD | 8FCA | 2023 | <https://www.nature.com/articles/s41467-023-39345-0#Sec21> |
| **TRPV4** | Human | --- |  | 8T1B | 2023 | <https://www.nature.com/articles/s41467-023-39345-0#Sec21> |
| **TRPV4** | Human | 4α-PDD | VSLD | 8T1D  8T1E | 2023 | <https://www.nature.com/articles/s41467-023-39345-0#Sec21> |
| **TRPV4** | Human | HC-067047 | VSLD | 8T1F | 2023 | <https://www.nature.com/articles/s41467-023-39345-0#Sec21> |
| **TRPV4** | Mouse | GSK101 | VSLD | 8J1F  8J1B |  | https://doi.org/10.1038/s41421-023-00579-3 |
| **TRPV4** | Mouse | --- |  | 8J1D |  | <https://doi.org/10.1038/s41421-023-00579-3> |
| **TRPV4** | Mouse | Agonist1 | VSLD | 8J1H |  | <https://doi.org/10.1038/s41421-023-00579-3> |
| **TRPV4** | Frog | Gd^3+^ |  | 6C8H | 2018 | <https://doi.org/10.1038/s41594-018-0037-5> |
| **TRPV4** | Frog | Cs+ |  | 6C8F | 2018 | <https://doi.org/10.1038/s41594-018-0037-5> |
| **TRPV4** | Frog | Ba2+ |  | 6C8G | 2018 | <https://doi.org/10.1038/s41594-018-0037-5> |
| **TRPV4** | Human | 4alpha-PDD | VSLD | 7AA5 | 2021 |  |
| **TRPV4** | Frog | --- |  | 6BBJ | 2018 | <https://doi.org/10.1038/s41594-018-0037-5> |
| **TRPV3** | Human | Trpvicin | Vanilloid Pocket | 7XJ0  7XJ1  7XJ2 | 2022 | <https://doi.org/10.1038/s41589-022-01166-5> |
| **TRPV3** | Mouse | Osthole | VSLD and pre-S1/S45-loop/TRP box interface | 7RAU 7RAS | 2021 | <https://doi.org/10.15252/embr.202153233> |
| **TRPV3** | Mouse | Dyclonine | portal site | 7UGG | 2022 | <https://doi.org/10.1038/s41467-022-30537-8> |
| **TRPV3** | Human | 2-APB | VSLD pocket | 6UW8 6UW9 | 2019 | <https://doi.org/10.1038/s41594-020-0428-2> |
| **TRPV3** | Human | --- |  | 6OT2 | 2019 | <https://doi.org/10.7554/eLife.47746> |
| **TRPV3** | Human | 2-APB | pre-S1/S45-loop/TRP box interface | 6OT5 | 2019 | <https://doi.org/10.7554/eLife.47746> |
| **TRPV3** | Mouse | 2-APB | VSLD and pre-S1/S45-loop/TRP helix interface | 6DVY | 2018 | <https://doi.org/10.1038/s41594-018-0108-7> |
| **TRPV3** | Mouse | 2-APB | VSLD and pre-S1/S45-loop/TRP helix interface | 6DVZ | 2018 | <https://doi.org/10.1038/s41594-018-0108-7> |
| **TRPV3** | Human | 2-APB | VSLD pocket | 6MHV 6MHW 6MHX | 2018 | <https://doi.org/10.1038/s41467-018-07117-w> |
| **TRPV3** | Human | --- |  | 8GKG  8KGA | 2023 | <https://doi.org/10.1038/s41586-023-06470-1> |
| **TRPV3** | Human | ---- |  | 6UW4  6UW6 | 2019 | <https://doi.org/10.1038/s41594-020-0428-2> |
| **TRPV3** | Human | --- |  | 7XJ3 | 2022 | <https://doi.org/10.1038/s41589-022-01166-5> |
| **TRPV3** | Mouse | --- |  | 7MIJ  7MIK  7MIL  7MIM  7MIN  7MIO | 2021 | <https://doi.org/10.1038/s41594-021-00615-4> |
| **TRPV3** | Mouse | --- |  | 6PVL  6PVM  6PVN  6PVO  6PVP  6PVQ | 2019 | <https://doi.org/10.1038/s41594-019-0318-7> |
| **TRPV3** | Mouse | --- |  | 6LGP | 2019 | <https://doi.org/10.1038/s41594-020-0439-z> |
| **TRPV3** | Mouse | --- |  | 6DVW | 2018 | <https://doi.org/10.1038/s41594-018-0108-7> |
| **TRPV3** | Human | --- |  | 6MHO  6MHS | 2018 | <https://doi.org/10.1038/s41467-018-07117-w> |
| **TRPV2** | Rabbit | RTX | Vanilloid Pocket | 6OO3 6OO4 6OO5 6OO7 | 2019 | <https://doi.org/10.7554/eLife.45779> |
| **TRPV2** | Rabbit | RTx | Vanilloid Pocket | 6BWJ | 2018 | <https://doi.org/10.1038/s41594-018-0059-z> |
| **TRPV2** | Rat | Piperlongumine (PL) | S45 site | 6WKN | 2020 | <https://doi.org/10.1021/acscentsci.1c00070> |
| **TRPV2** | Rat | phytocannabinoid tetrahydrocannabiorcol (C16), Probenecid (Pro), both |  | 7ZJE  7ZJG  7ZJH | 2022 | <https://doi.org/10.1038/s41467-022-35163-y> |
| **TRPV2** | Mouse | --- |  | 7XEO  7XER  7XEW | 2022 | <https://doi.org/10.1038/s41589-022-01139-8> |
| **TRPV2** | Mouse | estradiol (E2) | vanilloid pocket (cholesterol derivative) | 7XEU | 2022 | https://doi.org/10.1038/s41589-022-01139-8 |
| **TRPV2** | Mouse | 2-APB | VSLD | 7XEV  7YEP | 2022 | <https://doi.org/10.1038/s41589-022-01139-8> |
| **TRPV2** | Mouse | Cholesterol | vanilloid pocket (cholesterol) (antagonist) | 7XEM | 2022 | <https://doi.org/10.1038/s41589-022-01139-8> |
| **TRPV2** | Rat | CBD, PI lipid | portal site | 8SLX 8SLY | 2023 | <https://doi.org/10.1101/2023.01.27.525817> |
| **TRPV2** | Rat | CBD | portal site | 6U88 6U8A | 2019 | <https://doi.org/10.7554/eLife.48792> |
| **TRPV2** | Rat | 2-APB and CBD | CBD: portal site; 2-APB: S45 site | 7T37 7T38 | 2021 | <https://doi.org/10.1038/s41467-022-30083-3> |
| **TRPV2** | Rat | 2-APB | S45 site | 7N0M  7N0N | 2021 | <https://doi.org/10.1038/s41467-022-30083-3> |
| **TRPV2** | Rat, virus | --- |  | 7ZJD  7ZJD | 2022 | <https://doi.org/10.1038/s41467-022-35163-y> |
| **TRPV2** | Rat | --- |  | 6BO4  6BO5 | 2019 | <https://doi.org/10.1038/s41594-018-0168-8> |
| **TRPV2** | Rat | --- |  | 6U84  6U86 | 2019 | <https://doi.org/10.7554/eLife.48792> |
| **TRPV2** | Rabbit | --- |  | 6BWM | 2018 | <https://doi.org/10.1038/s41594-018-0059-z> |
| **TRPV2** | Rabbit | --- |  | 5AN8 | 2016 | <https://doi.org/10.1038/nsmb.3159> |
| **TRPV2** | Rat | --- |  | 5HI9 | 2016 | <https://doi.org/10.1038/ncomms11130> |
| **TRPV1** | Human | SB-366791 | center pore site | 8GFA | 2013 | <https://doi.org/10.1038/s41467-023-38162-9> |
| **TRPV1** | Rat | RTX, NMDG | Vanilloid Pocket | 7L2V 7L2W 7L2X | 2021 | <https://doi.org/10.1016/j.cell.2021.08.012> |
| **TRPV1** | Rat | RTX | Vanilloid Pocket | 7L2L 7L2N 7L2O | 2021 | <https://doi.org/10.1016/j.cell.2021.08.012> |
| **TRPV1** | Rat | DkTx/RTX | extracellular | 7L2M | 2021 | <https://doi.org/10.1016/j.cell.2021.08.012> |
| **TRPV1** | Rat | DkTx | extracellular | 7L2R  7L2S  7L2T  7L2U | 2021 | <https://doi.org/10.1016/j.cell.2021.08.012> |
| **TRPV1** | Squirrel | RTX | Vanilloid Pocket | 7LQZ | 2021 | <https://doi.org/10.1038/s41467-021-22507-3> |
| **TRPV1** | Rat | RTX | Vanilloid Pocket | 7MZ5 7MZ6 7MZ7 7MZ9 7MZA 7MZB  7MZC 7MZE | 2021 | <https://doi.org/10.1016/j.cell.2021.08.012> |
| **TRPV1** | Rat | RTX | Vanilloid Pocket | 7RQU 7RQV 7RQW 7RQX 7RQY 7RQZ | 2021 | <https://doi.org/10.1038/s41467-022-30602-2> |
| **TRPV1** | Rat, spider | DkTx and RTX | extracellular | 5IRX | 2016 | <https://doi.org/10.1038/nature12823> |
| **TRPV1** | Rat | DkTx and RTX | extracellular | 3J5Q | 2013 | <https://doi.org/10.1038/nature12823> |
| **TRPV1** | Rat | Capsaicin | Vanilloid pocket | 3J5R | 2013 | <https://doi.org/10.1038/nature12823> |
| **TRPV1** | Rat | Capsazepine | Vanilloid pocket | 5IS0 | 2016 | <https://doi.org/10.1038/nature12823> |
| **TRPV1** | Squirrel | Capsaicin | Vanilloid pocket | 7LR0 | 2021 | <https://doi.org/10.1038/s41467-021-22507-3> |
| **TRPV1** | Rat | Capsaicin | Vanilloid pocket | 7LPA 7LPB 7LPC 7LPD 7LPE | 2021 | <https://doi.org/10.1038/s41594-021-00616-3> |
| **TRPV1** | Rat | --- |  | 7L2H 7L2I  7L2J 7L2K | 2021 | <https://doi.org/10.1016/j.cell.2021.08.012> |
| **TRPV1** | Squirrel | --- |  | 7LQY | 2021 | <https://doi.org/10.1038/s41467-021-22507-3> |
| **TRPV1** | Rat | --- |  | 7LP9 | 2021 | <https://doi.org/10.1038/s41594-021-00616-3> |
| **TRPV1** | Rat | --- |  | 5IRZ | 2016 | <https://doi.org/10.1038/nature12823> |
| **TRPV1** | Rat | --- |  | 3J5P | 2013 | <https://doi.org/10.1038/nature12822> |
| **TRPV1** | Human | --- |  | 8GF8  8GF9 | 2013 | <https://doi.org/10.1038/s41467-023-38162-9> |
| **TRPV1** | Rat |  |  | 7L2P | 2021 | <https://doi.org/10.1016/j.cell.2021.08.012> |
| **TRPM8** | Bird | TC-I 2014 | VSLD pocket | 6O72 | 2019 | <https://doi.org/10.1126/science.aax6672> |
| **TRPM8** | Bird | PI(4,5)P2, WS-12 | PIP2: pre-S1, MHR4 & S4-5 loop;  WS-12: VSLD pocket | 6NR2 | 2019 | <https://doi.org/10.1126/science.aav9334> |
| **TRPM8** | Mouse | PI(4,5)P2 (putative) |  | 8E4N  8E4O | 2022 | <https://doi.org/10.1126/science.add1268> |
| **TRPM8** | Bird | PI(4,5)P2 | pre-S1, MHR4 & S4-5 loop | 8E4Q | 2022 | <https://doi.org/10.1126/science.add1268> |
| **TRPM8** | Bird | icilin, PI(4,5)P2, Ca2+ | PIP2: pre-S1, MHR4 & S4-5 loop;  Icilin: VSLD pocket | 6NR4  6NR3 | 2019 | <https://doi.org/10.1126/science.aav9334> |
| **TRPM8** | Mouse | cryosim-3 (C3), PI(4,5)P2 | PIP2: pre-S1, MHR4 & S4-5 loop;  cryosim-3 in VSLD pocket | 8E4M | 2022 | <https://doi.org/10.1126/science.add1268> |
| **TRPM8** | Mouse | cryosim-3 (C3), AITC, PI(4,5)P2 | PIP2: pre-S1, MHR4 & S4-5 loop;  C3: VSLD pocket | 8E4L | 2022 | <https://doi.org/10.1126/science.add1268> |
| **TRPM8** | Mouse | Ca^2+^, PI(4,5)P2, icilin | PIP2: pre-S1, MHR4 & S4-5 loop;  Icilin: VSLD pocket | 7WRC 7WRF | 2022 | <https://doi.org/10.1038/s41467-022-30919-y> |
| **TRPM8** | Mouse | Ca^2+^, icilin | VSLD pocket | 7WRD 7WRE | 2022 | <https://doi.org/10.1038/s41467-022-30919-y> |
| **TRPM8** | Mouse | Ca^2+^ |  | 7WRB | 2022 | <https://doi.org/10.1038/s41467-022-30919-y> |
| **TRPM8** | Bird | Ca^2+^ |  | 6O77 | 2019 | <https://doi.org/10.1126/science.aax6672> |
| **TRPM8** | Bird | AMTB | VSLD pocket | 6O6R | 2019 | <https://doi.org/10.1126/science.aax6672> |
| **TRPM8** | Mouse | --- |  | 7WRA | 2022 | <https://doi.org/10.1038/s41467-022-30919-y> |
| **TRPM8** | Mouse | --- |  | 8E4P | 2022 | <https://doi.org/10.1126/science.add1268> |
| **TRPM8** | Bird | --- |  | 6BPQ | 2017 | <https://doi.org/10.1126/science.aan4325> |
| **TRPM8** | Bird | --- |  | 6O6A | 2019 | <https://doi.org/10.1126/science.aax6672> |
| **TRPM7** | Mouse | VER155008 | Vanilliod-like pocket | 8SI8 | 2023 | <https://doi.org/10.1038/s41467-023-38362-3> |
| **TRPM7** | Mouse | naltriben | Vanilliod-like pocket | 8SI6 | 2023 | <https://doi.org/10.1038/s41467-023-38362-3> |
| **TRPM7** | Mouse | naltriben | MHR4/Pre-S1 interface | 8SI5 | 2023 | <https://doi.org/10.1038/s41467-023-38362-3> |
| **TRPM7** | Mouse | GDN, VER155008 | Vanilliod-like pocket | 8SI7 | 2023 | <https://doi.org/10.1038/s41467-023-38362-3> |
| **TRPM7** | Mouse | GDN, NS8593 | Vanilliod-like pocket | 8SIA | 2023 | <https://doi.org/10.1038/s41467-023-38362-3> |
| **TRPM7** | Mouse | GDN |  | 8SI3  8SI4 | 2023 | <https://doi.org/10.1038/s41467-023-38362-3> |
| **TRPM7** | Mouse | EDTA |  | 5ZX5 | 2018 | <https://doi.org/10.1073/pnas.1810719115> |
| **TRPM7** | Mouse | --- |  | 6BWF  6BWD | 2018 | <https://doi.org/10.1073/pnas.1810719115> |
| **TRPM7** | Mouse | --- |  | 8SI2  8SIB | 2023 | <https://doi.org/10.1038/s41467-023-38362-3> |
| **TRPM7** | Rat | --- |  | 3E7K | 2008 | <https://doi.org/10.1016/j.jmb.2008.08.059> |
| **TRPM5** | Zebrafish | EDTA |  | 7MBP | 2021 | <https://doi.org/10.1038/s41594-021-00607-4> |
| **TRPM5** | Zebrafish | Ca^2+^ and NDNA | Vanilliod-like pocket | 7MBV | 2021 | <https://doi.org/10.1038/s41594-021-00607-4> |
| **TRPM5** | Zebrafish | Ca^2+^ |  | 7MBR 7MBS  7MBQ  7MBT  7MBU | 2021 | <https://doi.org/10.1038/s41594-021-00607-4> |
| **TRPM4** | Human | Ca^2+^ and decavanadate |  | 5WP6 | 2017 | <https://doi.org/10.1038/nature24674> |
| **TRPM4** | Human | Ca^2+^ |  | 6BQV | 2018 | <https://doi.org/10.1126/science.aar4510> |
| **TRPM4** | Mouse | ATP | cytosolic ATP-binding site | 6BCO 6BCQ | 2017 | <https://doi.org/10.1038/nature24997> |
| **TRPM4** | Mouse | --- |  | 6BCJ  6BCL | 2017 | <https://doi.org/10.1038/nature24997> |
| **TRPM4** | Human | --- |  | 6BQR | 2018 | <https://doi.org/10.1126/science.aar4510> |
| **TRPM4** | Human | --- |  | 6BWI | 2017 | <https://doi.org/10.1073/pnas.1722038115> |
| **TRPM3** | Mouse | PIP2, PregS | pre-S1/S45 loop/TRP interface | 8ED8  8ED9 | 2022 | <https://doi.org/10.1016/j.neuron.2022.10.002> |
| **TRPM3** | Mouse | PIP2 | pre-S1/S45 loop/TRP interface | 8DDS 8DDT  8DDU  8DDV | 2022 | <https://doi.org/10.1016/j.neuron.2022.10.002> |
| **TRPM3** | Mouse, Human | Gbg, PIP2, ALFA-nanobody tether | pre-S1/S45 loop/TRP interface | 8DDX | 2022 | <https://doi.org/10.1016/j.neuron.2022.10.002> |
| **TRPM3** | Mouse, Human | Gbg, ALFA-nanobody tether |  | 8DDW | 2022 | <https://doi.org/10.1016/j.neuron.2022.10.002> |
| **TRPM3** | Mouse | Gbg |  | 8DDQ | 2022 | <https://doi.org/10.1016/j.neuron.2022.10.002> |
| **TRPM3** | Mouse | --- |  | 8ED7 | 2022 | <https://doi.org/10.1016/j.neuron.2022.10.002> |
| **TRPM3** | Mouse | --- |  | 8DDR | 2022 | <https://doi.org/10.1016/j.neuron.2022.10.002> |
| **TRPM2** | Starfish | Ca^2+^ |  | 6CO7 | 2018 | <https://doi.org/10.7554/eLife.36409> |
| **TRPM2** | Zebrafish | Ca^2+^ |  | 6D73 | 2018 | <https://doi.org/10.1038/s41467-019-11733-5> |
| **TRPM2** | Zebrafish | ADPR, Ca2+ | cytosolic ADPR binding site | 6PKX | 2018 | <https://doi.org/10.1038/s41467-019-11733-5> |
| **TRPM2** | Zebrafish | ADPR, Ca2+ |  | 6DRJ | 2018 | <https://doi.org/10.1038/s41586-018-0558-4> |
| **TRPM2** | Human | ADPR and Ca^2+^ |  | 6PUS | 2019 | <https://doi.org/10.7554/eLife.50175> |
| **TRPM2** | Human | ADPR and Ca^2+^ |  | 6MJ2 | 2018 | <https://doi.org/10.1126/science.aav4809> |
| **TRPM2** | Human | ADPR |  | 6PUR | 2019 | <https://doi.org/10.7554/eLife.50175> |
| **TRPM2** | Human | ADPR |  | 6MIZ | 2018 | <https://doi.org/10.1126/science.aav4809> |
| **TRPM2** | Human | 8-Br-cADPR, Ca^2+^ | cytosolic ADPR binding site | 6PUU | 2019 | <https://doi.org/10.7554/eLife.50175> |
| **TRPM2** | Human | ---- |  | 7VQ1  7VQ2 | 2021 | <https://doi.org/10.1016/j.celrep.2021.110025> |
| **TRPM2** | Zebrafish | --- |  | 6PKV 6PKW | 2018 | <https://doi.org/10.1038/s41467-019-11733-5> |
| **TRPM2** | Zebrafish | --- |  | 7AOV | 2022 | <https://doi.org/10.1002/pro.4320> |
| **TRPM2** | Human | --- |  | 6PUO | 2019 | <https://doi.org/10.7554/eLife.50175> |
| **TRPM2** | Human | --- |  | 6MIX | 2018 | <https://doi.org/10.1126/science.aav4809> |
| **TRPM2** | Zebrafish |  |  | 6DRK | 2018 | <https://doi.org/10.1038/s41586-018-0558-4> |
| **TRPC6** | Human | SAR7334 | VSLD pocket | 7DXG | 2022 | <https://doi.org/10.1016/j.neuron.2021.12.023> |
| **TRPC6** | Human | BTDM | vanilloid-like pocket | 7DXF | 2022 | <https://doi.org/10.1016/j.neuron.2021.12.023> |
| **TRPC6** | Human | AM-1473 | VSLD pocket | 6UZA | 2019 | <https://doi.org/10.7554/eLife.53311> |
| **TRPC6** | Human | AM-0883 | TM binding pocket (subunit interface formed by S6 and pore helix) | 6UZ8 | 2019 | <https://doi.org/10.7554/eLife.53311> |
| **TRPC6** | Human | --- |  | 5YX9 | 2017 | <https://doi.org/10.1038/s41422-018-0038-2> |
| **TRPC6** | Mouse | --- |  | 6CV9 | 2018 | <https://doi.org/10.1074/jbc.RA118.003183> |
| **TRPC6** | Human | --- |  | 7A6U | 2020 | <https://doi.org/10.1074/jbc.RA118.003183> |
| **TRPC5** | Human | riluzole | VSLD pocket | 7WDB | 2021 | <https://doi.org/10.1038/s41421-022-00410-5> |
| **TRPC5** | Human | HC-608 | TM binding pocket (subunit interface formed by S6 and pore helix & S5 from neighbor) | 6YSN | 2020 | <https://doi.org/10.1038/s42003-020-01437-8> |
| **TRPC5** | Human | HC-070 | TM binding pocket (subunit interface formed by S6 and pore helix & S5 from neighbor) | 7D4Q | 2020 | <https://doi.org/10.7554/eLife.63429> |
| **TRPC5** | Human | Clemizole | VSLD pocket | 7D4P | 2020 | <https://doi.org/10.7554/eLife.63429> |
| **TRPC5** | Mouse | --- |  | 6AEI | 2018 | <https://doi.org/10.1126/sciadv.aaw7935> |
| **TRPC5** | Human | --- |  | 7E4T | 2020 | <https://doi.org/10.7554/eLife.63429> |
| **TRPC5** | Human | --- |  | 7X6C 8GVW | 2022 | <https://doi.org/10.1038/s41467-023-38281-3> |
| **TRPC5** | Human | --- |  | 7XCI  8GVX | 2022 | <https://doi.org/10.1038/s41467-023-38281-3> |
| **TRPC4** | Zebrafish | --- |  | 7B0J | 2020 | <https://doi.org/10.7554/eLife.60603> |
| **TRPC4** | Zebrafish | --- |  | 6G1K | 2018 | <https://doi.org/10.7554/eLife.36615> |
| **TRPC4** | Mouse | --- |  | 5Z96 | 2018 | <https://doi.org/10.1038/s41467-018-05247-9> |
| **TRPC4** | Mouse | --- |  | 6JZO | 2020 | <https://doi.org/10.1038/s41467-018-05247-9> |
| **TRPC4** | Mouse | Calmodulin_Nlobe |  | 7CQP | 2020 | <https://doi.org/10.1016/j.str.2020.11.016> |
| **TRPC4** | Fruitfly | Calmodulin_Nlobe |  | 7CQV | 2020 | <https://doi.org/10.1016/j.str.2020.11.016> |
| **TRPC4** | Fruitfly | Calmodulin_Clobe |  | 7CQH | 2020 | <https://doi.org/10.1016/j.str.2020.11.016> |
| **TRPC4** | Zebrafish, Mouse | Calmodulin |  | 7B1G | 2020 | <https://doi.org/10.7554/eLife.60603> |
| **TRPC4** | Zebrafish | GFB-9289 | VSLD pocket | 7B16 | 2020 | <https://doi.org/10.7554/eLife.60603> |
| **TRPC4** | Zebrafish | GFB-8749 | VSLD pocket | 7B05 | 2020 | <https://doi.org/10.7554/eLife.60603> |
| **TRPC4** | Zebrafish | GFB-8438 | VSLD pocket | 7B0S | 2020 | <https://doi.org/10.7554/eLife.60603> |
| **TRPC3** | Human | lipid-occupied | a lipid (FGJ): TM binding pocket; 6OE: pre-S1/S45 loop/TRP interface | 6CUD | 2018 | <https://doi.org/10.7554/eLife.36852> |
| **TRPC3** | Human | high, low Ca^2+^ |  | 7DXB 7DXC | 2022 | <https://doi.org/10.1016/j.neuron.2021.12.023> |
| **TRPC3** | Human | --- |  | 6DJR  6DJS  6D7L | 2018 | <https://doi.org/10.1074/jbc.RA118.005066> |
| **TRPC3** | Human | --- |  | 7DXD  7DXD | 2022 | <https://doi.org/10.1016/j.neuron.2021.12.023> |
| **TRPC3** | Human | --- |  | 5ZBG | 2018 | <https://doi.org/10.1038/s41422-018-0038-2> |
|  |  |  |  |  |  |  |
| **TRPA1** | Human | JT010 | coupling domain pocket | 6PQO | 2019 | <https://doi.org/10.1016/j.neuron.2019.11.023> |
| **TRPA1** | Human | BITC | coupling domain pocket | 6PQP | 2019 | <https://doi.org/10.1016/j.neuron.2019.11.023> |
| **TRPA1** | Human | --- |  | 6PQQ | 2019 | <https://doi.org/10.1016/j.neuron.2019.11.023> |
| **TRPA1** | Human | Ca^2+^ | VSLD pocket | 6V9V  6V9W | 2019 | <https://doi.org/10.1038/s41586-020-2480-9> |
| **TRPA1** | Human | PMAL-C8 | small molecule not resolved | 6V9X | 2019 | <https://doi.org/10.1038/s41586-020-2480-9> |
| **TRPA1** | Human | PMAL-C8, A-967079 |  | 6V9Y | 2019 | <https://doi.org/10.1038/s41586-020-2480-9> |
| **TRPA1** | Human | GDC-0334 | TM binding pocket | 6WJ5 | 2020 | <https://doi.org/10.1084/jem.20201637> |
| **TRPA1** | Human | Compound 21 | pre-S1/S45 loop/TRP helix interface | 7JUP | 2021 | <https://doi.org/10.1021/acs.jmedchem.0c02023> |
| **TRPA1** | Human | GNE551 | vanilloid-like pocket | 6X2J | 2021 | <https://doi.org/10.1016/j.neuron.2020.10.014> |
| **TRPA1** | Human | --- |  | 3J9P | 2015 | <https://doi.org/10.1038/nature14367> |
| **TRPA1** | Human | 3-60 | pre-S1/S45 loop/TRP helix interface | 7OR0  7OR1 | 2021 | To be published |
| **TRPA1** | Human | FIH, Zn, NOG |  | 6HC8 | 2018 | To be published |
|  |  |  |  |  |  |  |
| **TRPA1** | Drosophila melanogaster | --- |  | 7YKR  7YKS | 2023 | To be published |
| **TRPML1** | Human | PI(4,5)P2 | VSLD pocket | 6E7Y | 2018 | <https://doi.org/10.1038/s41467-018-06493-7> |
| **TRPML1** | Mouse | PI(3,5)P2, temsirolimus | PIP2: VSLD pocket; temsirolimus: portal site | 7SQ9 | 2021 | <https://doi.org/10.1073/pnas.2120404119> |
| **TRPML1** | Human | PI(3,5)P2 and ML-SA1 | PIP2: VSLD pocket;  ML-SA1: portal site | 6E7Z | 2018 | <https://doi.org/10.1038/s41467-018-06493-7> |
| **TRPML1** | Mouse | PI(3,5)P2 | VSLD pocket | 7SQ7 | 2021 | <https://doi.org/10.1073/pnas.2120404119> |
| **TRPML1** | Human | PI(3,5)P2 | VSLD pocket | 6E7P | 2018 | <https://doi.org/10.1038/s41467-018-06493-7> |
| **TRPML1** | Human | ML-SI3 | portal site | 7MGL | 2021 | <https://doi.org/10.1016/j.str.2021.06.003> |
| **TRPML1** | Mouse | ML-SA1 | portal site | 7SQ6 | 2021 | <https://doi.org/10.1073/pnas.2120404119> |
| **TRPML1** | Human | ML-SA1 | portal site | 5WJ9 | 2017 | <https://doi.org/10.1038/nature24036> |
| **TRPML3** | Human | ML-SA1 | portal site | 6AYF | 2017 | <https://doi.org/10.1038/nsmb.3502> |
| **TRPML1** | Mouse | --- |  | 7SQ8 | 2021 | <https://doi.org/10.1073/pnas.2120404119> |
| **TRPML1** | Mouse | --- |  | 5YDZ  5YE1  5YE2  5YE5 | 2017 | <https://doi.org/10.1007/s13238-017-0476-5> |
| **TRPML1** | Human | --- |  | 5TJA  5JTB  5JTC | 2017 | <https://doi.org/10.1038/nsmb.3362> |
| **TRPML1** | Mouse | --- |  | 5WPQ  5WPT | 2017 | <https://doi.org/10.1038/nature24035> |
| **TRPML1** | Mouse | --- |  | 5WPV | 2017 | <https://doi.org/10.1038/nature24035> |
| **TRPML1** | Human | --- |  | 5WJ5 | 2017 | <https://doi.org/10.1038/nature24036> |
| **TRPML2** | Mouse | --- |  | 7DYS | 2021 | <https://doi.org/10.1016/j.jbc.2021.101487> |
| **TRPML2** | Human | --- |  | 6HRS  6HRR | 2019 | <https://doi.org/10.1016/j.str.2019.04.016> |
| **TRPML3** | Human | --- |  | 6AYE  6AYG | 2017 | <https://doi.org/10.1038/nsmb.3502> |
| **TRPML3** | Monkey | --- |  | 5W3S | 2017 | <https://doi.org/10.1038/nature24055> |
| **TRPP2 (PKD2)** | Human | --- |  | 5K47 | 2016 | <https://doi.org/10.1038/nsmb.3343> |
| **TRPP2**  **(PKD2)** | Human | --- |  | 5T4D | 2016 | <https://doi.org/10.1016/j.cell.2016.09.048> |
| **TRPP2 (PKD2)** | Human | PI(4,5)P2 | Vanilloid-like pocket | 6T9N | 2019 | <https://doi.org/10.1016/j.str.2019.11.005> |
| **TRPP2 (PKD2)** | Human | PI(3,5)P2 | Vanilloid-like pocket | 6T9O | 2019 | <https://doi.org/10.1016/j.str.2019.11.005> |
| **TRPP2 (PKD2)** | Human | Ca^2+^ |  | 5MKE 5MKF | 2017 | <https://doi.org/10.1038/nsmb.3357> |
| **TRPP2 (PKD2)** | Human | --- |  | 6D1W | 2018 | <https://doi.org/10.1038/s41467-018-04586-x> |
| **TRPP2 (PKD2)** | Human | --- |  | 6WB8 | 2020 | <https://doi.org/10.1073/pnas.1920777117> |
| **TRPP3 complex (PKD1L3/PKD2L1)** | Mouse | --- |  | 7D7E | 2020 | <https://doi.org/10.1038/s41467-021-25216-z> |
| **TRPP3 complex (PKD1L3/PKD2L1)** | Mouse | Ca^2+^ |  | 7D7F | 2020 | <https://doi.org/10.1038/s41467-021-25216-z> |
| **TRPP3 (PKD2L1)** | Mouse | --- |  | 5Z1W | 2017 | <https://doi.org/10.1038/s41467-018-03606-0> |
